# Low cholesterol is not associated with depression: data from the 2005-2018 National Health and Nutrition Examination Survey

**DOI:** 10.1186/s12944-022-01645-7

**Published:** 2022-04-03

**Authors:** Qun Zhang, Ziping Liu, Qian Wang, Xiaoqian Li

**Affiliations:** 1 Department of Medical Psychology, the Air Force Hospital of Northern Theater PLA, 46 Xiaoheyan Road, Shenyang, 110000 Liaoning China; 2 Department of Radiology, the First People’s Hospital of Xianyang, 10 Biyuan Road, Xianyang, 712000 Shaanxi China

**Keywords:** Depression, Low cholesterol, NHANES

## Abstract

**Background:**

Although high serum cholesterol is widely recognized as a major risk factor for heart disease, the health effects of low cholesterol are less clear. Several studies have found a correlation between low cholesterol and depression, but the results are inconsistent.

**Methods:**

Data from the National Health and Nutrition Examination Survey (NHANES) 2005-2018 were utilized in this cross-sectional study. The analysis of the relationship between cholesterol and depression was performed at three levels: low total cholesterol, low high-density lipoprotein (HDL) cholesterol and low-density lipoprotein (LDL) cholesterol. The inclusion criteria were as follows: (1) people with low (<4.14 mmol/L) or normal (4.14-5.16 mmol/L) total cholesterol for Sample 1; people with low (<1 mmol/L) or normal (≥1 mmol/L) HDL cholesterol levels for Sample 2; and people with low (<1.8 mmol/L) or normal (1.8-3.4 mmol/L) LDL cholesterol levels for Sample 3; and (2) people who completed the Patient Health Questionnaire-9 depression scale. Age, sex, educational level, race, marital status, self-rated health, alcohol status, smoking status, body mass index (BMI), poverty income ratio, physical function, comorbidities, and prescription use were considered potential confounders. The missing data were handled by multiple imputations of chained equations. Logistic regression was used to assess the relationship between low cholesterol and depression.

**Results:**

After controlling for potential confounding factors in the multivariate logistic regression, no association was observed between depression and low total cholesterol (OR=1.0, 95% CI: 0.9-1.2), low LDL cholesterol (OR=1.0, 95% CI: 0.8-1.4), or low HDL cholesterol (OR=0.9, 95% CI: 0.8-1.1). The results stratified by sex also showed no association between low total cholesterol, low LDL cholesterol, low HDL cholesterol and depression in either men or women.

**Conclusion:**

This population-based study did not support the assumption that low cholesterol was related to a higher risk of depression. This information may contribute to the debate on how to manage people with low cholesterol in clinical practice.

**Supplementary Information:**

The online version contains supplementary material available at 10.1186/s12944-022-01645-7.

## Background

Depression is a common but serious illness defined by persistent sadness and a loss of interest in things that one generally enjoys doing for at least two weeks [1]. The World Health Organization (WHO) reported that approximately 4.4% of the world’s population suffers from depression. In terms of lost health, the consequences of depression are substantial. The WHO estimates that depression is a leading cause of disease burden and disability, with data showing that depression accounts for 7.5% of all years lived with disability and is the single largest contributor to disability [2]. Depression is also the primary cause of deaths by suicide, with nearly 800 000 per year [2].

Normal cholesterol concentration is essential for receptor function, synaptic plasticity, and myelin formation in the central nervous system [3–7]. Given that the association is well demonstrated between neuronal deficits and depression [8, 9], a hypothesis has emerged that the link between depression and low cholesterol may have a neurobiological mechanism [3]. Several studies have reported that lowering cholesterol led to an increase in death rates due to other reasons, such as murder, accidents, and suicide, which offset the benefits of reducing heart disease [10–14]. Because depression is an important risk factor for suicide and fatal accidents, an idea has emerged that there is a link between depression and low cholesterol, based on both biological and clinical evidence [15].

Several studies have further investigated the connection between low cholesterol levels and depression, but the conclusions are contradictory. On the one hand, several studies [15–18] described that low cholesterol was correlated with a higher risk of depression. However, some studies reported that no significant association was discovered between depression and low cholesterol [19, 20]. The small sample sizes, sampling bias, and limited controls for confounding might be the most important reasons for the different conclusions.

The lack of consensus regarding a conclusion of the inconsistencies and the important clinical meaning created a need for further exploration of the association. This study aimed to identify the relationship between low cholesterol and depression based on the National Health and Nutrition Examination Survey (NHANES) dataset, with a large sample size representing the population of the United States and controls for confounding factors. In addition, associations were examined separately by sex to explore the sex differences in these relationships.

## Methods

### Design and study population

The data from the NHANES 2005-2018 were utilized in this cross-sectional study. The NHANES is a publicly available database, with a representative sample of the United States population [21]. To produce reliable statistics, the NHANES selected participants by a stratified multistage probability design, and certain racial, age, and income groups were oversampled.

Participants in the NHANES (2005–2018) were included in this study. The analysis was performed at three levels: low total cholesterol, low-density lipoprotein (LDL) cholesterol, and high-density lipoprotein (HDL) cholesterol. Because total cholesterol, LDL cholesterol, and HDL cholesterol were not examined in the same group of people, the analysis was based on three different samples. Sample 1 was selected for the analysis of total cholesterol, Sample 2 for HDL cholesterol, and Sample 3 for LDL cholesterol. The inclusion criteria were as follows: (1) people with low (<4.14 mmol/L) or normal (4.14-5.16 mmol/L) cholesterol levels were included in Sample 1; people with low (<1 mmol/L) or normal (≥1 mmol/L) HDL cholesterol levels were included in Sample 2; and people with low (<1.8 mmol/L) or normal (1.8-3.4 mmol/L) LDL cholesterol levels were included in Sample 3; and (2) people who completed the Patient Health Questionnaire-9 (PHQ-9). The exclusion criteria were (1) people with a total cholesterol level >5.16 mmol/L for sample 1 and those with an LDL cholesterol level >5.16 mmol/L for sample 3; and (2) people who did not have data for the PHQ-9.

### Depression

Depression was evaluated by the PHQ–9, a 9-item validated questionnaire evaluating depressive symptoms within the last 2 weeks [22]. The PHQ-9 classifies depressive symptoms into four levels (nearly every day; more than half the days; several days; and not at all). A score of 10 or higher was used to identify subjects with major depressive disorder (MDD). This clinical cutoff has a specificity and sensitivity of 88% in the detection of MDD [22]. The questionnaire was completed at a mobile exam center, and trained interviewers asked the questions using a computer-assisted personal interview system.

### Cholesterol

Blood specimens were analyzed at the University of Minnesota. Cholesterol levels were measured by an enzymatic assay: cholesterol esterase converted esterified cholesterol to cholesterol, which then produced hydrogen peroxide and cholest-4-en-3-one. After reacting with 4-aminophenazone by peroxidase, hydrogen peroxide then produced a colored product. This method is specific for cholesterol because it is a single reagent endpoint reaction. In this study, the total cholesterol levels were classified into a normal group (4.14-5.16 mmol/L) and a low group (<4.14 mmol/L) [15, 19]. LDL cholesterol levels were classified as normal (1.8-3.4 mmol/L) and low (<1.8 mmol/L) [23, 24]. HDL cholesterol levels were also classified as normal (≥1 mmol/L) and low (<1 mmol/L) [25].

### Potential confounder variables

Several potential confounding variables were taken into account. Age was divided into 18-39, 40-59, and >60 years. Race was classified as Mexican American, Non-Hispanic White, Non-Hispanic Black, other Hispanic, and other race. Marital status was classified as living with partner or married, widowed or divorced, never married, and separated. Educational level was divided into less than 9th grade, 9-11th grade, high school graduate, some college or AA degree, college graduate or above. Smoking status and alcohol status were classified as current, former, and never. Body mass index (BMI) was divided into overweight (i.e., ≥25), low (i.e., <18.5), and normal (i.e., 18.5-25). Self-rated health was divided into excellent, very good, good, fair, or poor. The poverty income ratio (PIR) was used to measure income, which was classified as poor (i.e., <1.0), nearly poor (i.e., 1.0-1.9), middle income (i.e., 2.0-3.9), and high income (i.e., ≥4.0). Prescription use for cholesterol was classified as “taking a prescription” if participants positively responded to the question “Now taking prescribed medicine” and classified as “no prescription” if they responded negatively to the questions “Now taking prescribed medicine” or “Ever had blood cholesterol checked”. Physical function was measured by six questions from the questionnaire that were consistent with previous literature [26], including (1) walking a quarter-mile; (2) lifting or carrying heavy objects; (3) standing from an armless chair; (4) climbing 10 steps; (5) stooping, crouching, or kneeling; and (6) standing for long periods [26]. If a person had any trouble performing these activities, they were considered to have an existing physical functioning limitation. The number of difficult problems was summarized on a scale of 0-6. Comorbidities were assessed using the question “Have you ever been told by a doctor or health professional that you have … ?” for the following comorbidities: high blood pressure, diabetes, stroke, cancer, and coronary heart disease.

### Data analysis

Stata 16.0 software was used to perform the analysis. The missing data were handled by multiple imputations of chained equations [27], and 6 imputed datasets were created using Stata’s mi impute. The variance estimation and weights were used to adjust for the complex survey method by Stata's svy. This study investigated the relationship between depression and low total cholesterol, low HDL cholesterol, and low LDL cholesterol in three different samples, and all statistical analyses were performed for each sample. Categorical data are described as percentages. The chi-square statistics were used in univariate analysis, and differences between groups were tested at the *P*<0.05 significance level. Then, three logistic regression models were calculated. Model 1 only included cholesterol, and no covariates were adjusted; Model 2 included cholesterol, age, and sex; Model 3 included all the covariates that were significant in the univariate analysis. Effect sizes were reported as odds ratios (ORs) with 95% confidence intervals (95% CIs). A *P* value of < 0.05 was considered statistically significant (two-sided).

## Results

**Fig. 1:**
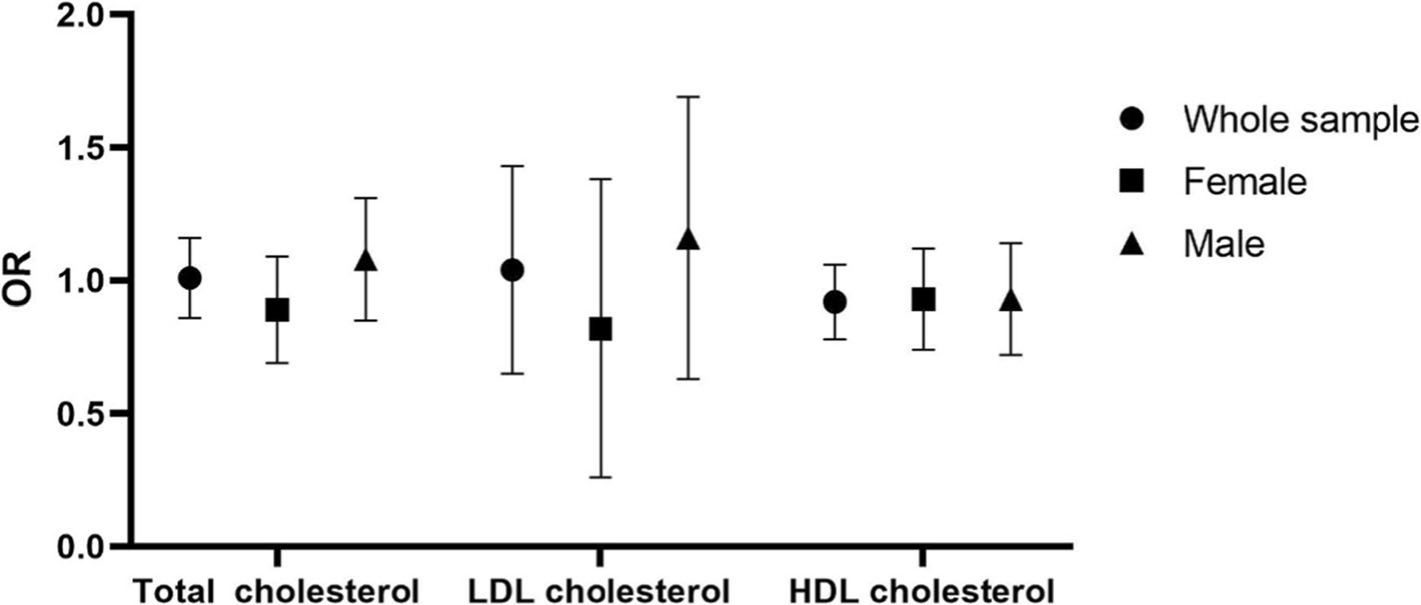
Association between low total cholesterol, low HDL-cholesterol, low LDL-cholesterol and depression

Figure 1 summarizes the results of logistic regression, and there was no association between low total cholesterol, low LDL cholesterol, low HDL cholesterol, and depression after adjustment for potential confounding factors in Model 3 for Sample 1, Sample 2, and Sample 3. The results were the same after stratification by sex in all three Samples.

For the analysis of total cholesterol in Sample 1, a total of 21550 subjects were included. Of these, there were 1809 people with depression (weighted proportion 7.5%) and 19,741 people without depression (weighted proportion 92.5%). Table 1 presents the characteristics of the weighted population. In the weighted sample, there was a significant difference in sex, race, educational level, family income, marital status, smoking status, alcohol status, BMI, physical function, coronary heart disease, cancer, stroke, high blood pressure, and diabetes (excluding prescription use). Therefore, these factors were added to the multivariate logistic regression model. No link was found between low total cholesterol levels and depression after adjustment for potential confounding factors (OR=1.0, 95% CI: 0.9-1.2). Then, the results were stratified by sex, and no link was found between low cholesterol levels and depression in either females (OR= 1.1, 95% CI: 0.9-1.3) or males (OR=0.9, 95% CI: 0.7-1.1) (Table 2).

**Table 1:** Weighted participant characteristics for total cholesterol in Sample 1

Characteristic	Depression	Non-depression	*P*
Sex (%)			<0.0001
Male	37.9	52.3	
Female	62.1	47.7	
Age (%)			0.0060
13-39	43.3	45.4	
40-59	34.8	29.8	
≥60	22.0	24.8	
Race (%)			0.0003
Mexican American	8.1	8.8	
Non-Hispanic White	7.0	5.4	
Non-Hispanic Black	62.9	67.0	
Other Hispanic	14.9	11.3	
Other Race	7.1	7.5	
Educational level (%)			<0.0001
Less than 9th grade	25.0	13.8	
9-11th grade	1.8	1.8	
High school graduate	28.1	23.4	
Some college or AA degree	34.4	31.6	
College graduate or above	10.8	29.6	
Poverty income ratio (%)			<0.0001
Poor	31.7	14.9	
Near-poor	28.4	20.6	
Middle-income	23.0	29.6	
High-income	16.9	34.9	
Marital status (%)			<0.0001
Married/Living with partner	46.7	61.3	
Divorced/Widowed	21.6	12.1	
Never married	24.4	24.1	
Separated	7.4	2.5	
Alcohol status (%)			<0.0001
Never	9.8	11.9	
Former	20.3	13.7	
Current	69.9	74.4	
Smoking status (%)			<0.0001
Never	39.1	59.9	
Former	21.7	21.6	
Current	39.2	18.5	
Health status (%)			<0.0001
Excellent	2.4	12.5	
Very good	11.3	34.8	
Good	34.2	39.0	
Fair	37.9	12.3	
Poor	14.2	1.5	
Body mass index (%)			0.0257
Low	2.3	2.1	
Normal	27.8	33.1	
Overweight	69.9	64.8	
Physical function score (%)			<0.0001
0	23.6	54.3	
1	14.3	15.6	
2	12.9	10.5	
3	12.8	7.9	
4	15.5	6.2	
5	11.9	3.5	
6	9.0	2.0	
Comorbidity Stroke (%)	6.6	2.9	<0.0001
Comorbidity coronary heart disease (%)	6.3	4.5	0.0124
Comorbidity cancer (%)	11.7	9.5	0.0437
Comorbidity high blood pressure (%)	41.5	29.9	<0.0001
Comorbidity diabetes (%)	15.4	10.7	0.0003
Take prescription for high cholesterol (%)	26.1	25.2	0.5242

**Table 2:** Association between low total cholesterol and depression before and after adjustment in Sample 1

	Model 1 OR (95% CI)	Model 2 OR (95% CI)	Model 3 OR (95% CI)
All sample	1.1 (1.0-1.2)	1.2 (1.0-1.3)*	1.0 (0.9-1.2)
Stratified by sex			
Male	1.0 (0.8-1.3)	1.1 (0.9-1.3)	0.9 (0.7-1.1)
Female	1.2 (1.0-1.4)*	1.1 (1.0-1.5)*	1.1 (0.9-1.3)

Model 1 only included total cholesterol, and no covariate was adjusted; Model 2 adjusted for age and sex; Model 3 adjusted for sex, age, race, educational level, marital status, smoking status, alcohol status, body mass index, self-rated health, poverty income ratio, physical function; high blood pressure; stroke; coronary heart disease; cancer, and diabetes. *P<0.05

For the analysis of HDL cholesterol in Sample 2, a total of 34459 subjects were included. Of these, there were 3006 people with depression (weighted proportion 7.7%) and 31453 people without depression (weighted proportion 92.3%). In the weighted sample, there was a significant difference in sex, race, educational level, family income, marital status, smoking status, alcohol status, BMI, physical function, diabetes, stroke, coronary heart disease, and high blood pressure (detailed information can be found in Supplemental Material, Table S1). Therefore, these factors were added to the multivariate logistic regression model. There was no relationship between low HDL cholesterol and depression in the whole group (OR=0.9, 95% CI: 0.8-1.1), in females (OR=1.0, 95% CI: 0.8-1.1), or in males (OR=0.9, 95% CI: 0.8-1.1) after adjusting for potential confounding factors (Table 3).

**Table 3:** Association between low HDL cholesterol and depression before and after adjustment in Sample 2

	Model 1 OR (95% CI)	Model 2 OR (95% CI)	Model 3 OR (95% CI)
All sample	1.2 (1.0-1.3)*	1.4 (1.2-1.6) *	0.9 (0.8-1.1)
Stratified by sex			
Male	1.2 (1.0-1.4)*	1.2 (1.0-1.4)*	0.9 (0.8-1.1)
Female	1.8 (1.5-2.1) *	1.7 (1.5-2.1)*	0.9 (0.8-1.1)

Model 1 only included HDL cholesterol, and no covariate was adjusted; Model 2 adjusted for age and sex; Model 3 adjusted for sex, age, race, educational level, marital status, smoking status, alcohol status, body mass index, self-rated health, poverty income ratio, physical function; high blood pressure; stroke; coronary heart disease; and diabetes. * *P* <0.05

For the analysis of LDL cholesterol in Sample 3, a total of 11141 subjects were included. Of these, there were 914 people with depression (weighted proportion 7.2%) and 10227 people without depression (weighted proportion 92.8%). In the weighted sample, there was a significant difference in sex, race, educational level, family income, marital status, smoking status, alcohol status, BMI, physical function, diabetes, high blood pressure, coronary heart disease, and stroke (detailed information can be found in Supplemental Material, Table S2). After adjustment, no association was found between low LDL cholesterol levels and depression in the whole group (OR=1.0, 95% CI: 0.8-1.4), in females (OR=1.2, 95% CI: 0.8-1.7), or in males (OR=0.8 95% CI: 0.5-1.4) (Table 4).

**Table 4:** Association between low LDL cholesterol and depression before and after adjustment in Sample 3

	Model 1 OR (95% CI)	Model 2 OR (95% CI)	Model 3 OR (95% CI)
All sample	1.1 (0.8-1.5)	1.3 (0.9-1.7)	1.0 (0.8-1.4)
Stratified by sex			
Male	1.0 (0.6-1.6)	1.0 (0.6-1.7)	0.8 (0.5-1.4)
Female	1.3 (0.9-1.8)	1.4 (1.0-2.0)	1.2 (0.8-1.7)

Model 1 only included LDL cholesterol, and no covariate was adjusted; Model 2 adjusted for age and sex; Model 3 adjusted for sex, age, race, educational level, marital status, smoking status, alcohol status, body mass index, self-rated health, poverty income ratio, physical function; high blood pressure; stroke; coronary heart disease; and diabetes

## Discussion

In this study, no association was discovered between low total cholesterol, low HDL cholesterol, low LDL cholesterol and depression in either males or females in a multivariate model that adjusted for potential confounding factors. This finding was consistent with several previous studies [19, 20].

Interestingly, some studies based on the NHANES or other cross-sectional studies have inconsistent conclusions. The differences in these results may be explained by at least three reasons that are discussed below.

First, the differences may be due to sampling error, as there are differences in the included populations in different studies. For instance, Morgan et al. reported that depression was more common in the low cholesterol subgroup than in the higher cholesterol subgroup in a sample of men aged 70 years or older [15]. Another case-control study with Mexican major depression patients showed that participants in the healthy control group had a high level of cholesterol (4.46±0.65 mmol/L), while those in the depression group had a low level (4.34±1.17 mmol/L) [17]. One study with middle-aged men reported that the low cholesterol (less than 4.5 mmol/L) group had a higher risk of depression than the high cholesterol (6-7 mmol/L) group [16].

Second, the differences may be explained by methodological differences. On the one hand, the confounding factors included were not the same. Actually, in a study based on the NHANES [18], the result of the unadjusted model was the same. On the other hand, the difference may be illustrated by the differences in how people were grouped. One study used low levels of cholesterol (<3.34 mmol/L) compared with higher levels (>6.18 mmol/L) [18]. In another study, depression was further divided into severe depression and moderate depression, and the intermediate group (4.40-5.74 mmol/L) was used as a reference for comparison with the low cholesterol group (<4.37 mmol/L) [28]. The results showed that low cholesterol was correlated with a higher risk of severe depression in males but not in males; low cholesterol levels were not correlated with moderate depression in either males or males. It is believed that compared with normal levels, rather high levels could be more helpful for clinical practice regarding low cholesterol, now that high cholesterol has been proven to be harmful.

Third, the potential biological mechanisms should be further discussed. Cholesterol is an important component of the central nervous system [3–6]; however, the majority of cholesterol in the brain is produced by de novo synthesis because of the blood-brain barrier. There was no clear evidence of a relationship between plasma and brain cholesterol levels [5, 29]. This means that the association between central nervous system cholesterol and depression is different from that between plasma cholesterol and depression. The specific relationship needs to be further explored.

For the use of lipid-lowering prescription drugs, no difference was found between the depression group and the non-depression group. Notably, proprotein convertase subtilisin-kexin type 9 (PSK-9) was found to be a key regulator of LDL cholesterol, and PSK-9 inhibition has shown excellent efficacy in decreasing LDL cholesterol [30]. The effect of PSK-9 on cognitive function and depression has been recently investigated in several studies [24, 31]. However, the NHANES only provided general prescription information for cholesterol, and this article could not determine the relationship between depression and the prescription of PSK-9 inhibitors.

In summary, this study did not support the assumption that low cholesterol was associated with depression. After adjusting for sex, age, race, education level, marital status, BMI, alcohol status, smoking status, self-rated health, poverty income ratio, physical function, comorbidities, and prescription use, no relationship was discovered between low cholesterol and a higher risk of depression in men or women. This information may contribute to the debate on how to manage people with low cholesterol.

### Comparisons with other studies and what does the current work add to the existing knowledge

Several studies have evaluated the relationship between cholesterol and depression [15–20, 28]. Cholesterol levels can be classified as low, normal, or high, and most previous studies combined the normal and high cholesterol groups as a new group for comparison with the low group. This comparison has some limitations. The value of investigating the link between low cholesterol and depression was mainly to provide advice for the following conditions: (1) whether people with naturally low cholesterol levels need to receive an active intervention to raise their cholesterol levels; (2) whether the risk of depression should be considered after offering advice on lipid-lowering drugs or diet; and (3) whether the recommendation of lowering cholesterol for primary prevention needs to be further considered for people who have a low risk of heart disease (that is, for patients with no previous cardiovascular events) [32]. If high cholesterol levels were included in the research, the conclusion would be influenced and clinical recommendations cannot be made for the above three conditions. In this study, the relationship between low cholesterol and depression was explored in a large population-based dataset, and a new classification method was used. This was the first time that groups with normal and low levels were compared. Therefore, this analysis provided better clinical value in clinical decision-making for the above three conditions.

### Strengths and limitations

There were several strengths in this study. First, this study used a population-based representative sample. Second, the normal cholesterol level was selected as the reference group for comparison with the low cholesterol level in this study, which was a novel classification method. Third, this topic is important for patients, and this study can be helpful in clinical practice. Several limitations also existed in this study. First, the analysis was based on a cross-sectional design, which cannot draw any causal inferences even after adjusting for some confounding factors. Second, patients with depression who have been successfully treated cannot be recognized by the PHQ-9. Further investigation of the mechanisms and longitudinal studies are required to explain these discoveries. Third, it is worth mentioning that the NHANES was designed as a cross-sectional study and does not have data about basal cholesterol or other laboratory values. Therefore, this study cannot determine the longitudinal relationship between low cholesterol and depression; this was the main limitation of this study.

## Conclusion

This population-based study did not support the assumption that low cholesterol was related to a higher risk of depression. This information may contribute to the debate on how to manage people with low cholesterol in clinical practice, as different studies have come to conflicting conclusions, with some reporting benefits of low cholesterol and others reporting harmful consequences. Based on this study, doctors do not need to be afraid to attempt to reduce cholesterol levels in patients with high levels due to the risk of depression. It is hoped that the potential improvement of the novel classification presented in this study will be useful in designing future studies of low cholesterol.

## Supplementary Information

**Additional file 1.**

## Data Availability

This datasets that were created and analyzed during this investigation can be found at https://www.cdc.gov/nchs/nhanes/index.htm.
