# Supplementary material for: Low cholesterol is not associated with depression: data from the 2005-2018 National Health and Nutrition Examination Survey
Source: Lipids Health Dis. 2022 Apr 3;21:35. doi: 10.1186/s12944-022-01645-7 (PMC8978383; doi:10.1186/s12944-022-01645-7)
Supplement: Supplementary file 1 — Additional file 1. [file 12944_2022_1645_MOESM1_ESM.docx]

Supplement Material

**Table 1. Weighted participant characteristics for HDL cholesterol in Sample 2**

| Characteristic | Depression | Non-depression | *P* |
| --- | --- | --- | --- |
| Gender (%) |  |  | <0.0001 |
| Male | 36.1 | 50.0 |  |
| Female | 63.8 | 50.0 |  |
| Age (%) |  |  | <0.0001 |
| 13-39 | 36.9 | 38.1 |  |
| 40-59 | 41.6 | 36.1 |  |
| ≥60 | 21.5 | 25.8 |  |
| Race (%) |  |  | <0.0001 |
| Mexican American | 8.5 | 8.7 |  |
| Non-Hispanic White | 63.3 | 68.4 |  |
| Non-Hispanic Black | 13.2 | 10.4 |  |
| Other Hispanic | 7.7 | 5.3 |  |
| Other Race | 7.3 | 7.2 |  |
| Educational level (%) |  |  | <0.0001 |
| Less than 9th grade | 24.5 | 14.2 |  |
| 9-11th grade | 0.9 | 1.0 |  |
| High school graduate | 27.4 | 23.5 |  |
| Some college or AA degree | 33.9 | 31.4 |  |
| College graduate or above | 13.3 | 29.9 |  |
| Poverty income ratio (%) |  |  | <0.0001 |
| Poor | 29.0 | 12.9 |  |
| Near-poor | 29.3 | 19.6 |  |
| Middle-income | 23.1 | 29.3 |  |
| High-income | 18.6 | 38.2 |  |
| Marital status (%) |  |  | <0.0001 |
| Married/Living with partner | 48.4 | 65.1 |  |
| Divorced/Widowed | 25.0 | 15.2 |  |
| Never married | 20.9 | 17.7 |  |
| Separated | 5.61 | 2.1 |  |
| Alcohol status (%) |  |  | <0.0001 |
| Never | 9.4 | 11.1 |  |
| Former | 21.9 | 14.9 |  |
| Current | 68.6 | 74.0 |  |
| Smoking status (%) |  |  | <0.0001 |
| Never | 38.6 | 56.6 |  |
| Former | 22.3 | 25.0 |  |
| Current | 39.0 | 18.4 |  |
| Health status (%) |  |  | <0.0001 |
| Excellent | 2.21 | 11.56 |  |
| Very good | 11.9 | 34.5 |  |
| Good | 35.4 | 39.6 |  |
| Fair | 36.4 | 12.9 |  |
| Poor | 14.1 | 1.4 |  |
| BMI (%) |  |  | 0.0015 |
| Low | 2.1 | 1.6 |  |
| Normal | 24.6 | 28.9 |  |
| Overweight | 73.2 | 68.6 |  |
| Physical function score (%) |  |  |  |
| 0 | 25.6 | 45.2 |  |
| 1 | 15.1 | 18.7 |  |
| 2 | 13.6 | 13.6 |  |
| 3 | 13.9 | 9.4 |  |
| 4 | 11.9 | 6.6 |  |
| 5 | 11.0 | 4.37 |  |
| 6 | 9.1 | 2.13 |  |
| Comorbidity Stroke (%) | 6.6 | 2.5 | <0.0001 |
| Comorbidity coronary heart disease (%) | 5.8 | 3.3 | <0.0001 |
| Comorbidity cancer (%) | 11.7% | 10.0 | 0.0582 |
| Comorbidity high blood pressure (%) | 42.7 | 30.3 | <0.0001 |
| Comorbidity diabetes (%) | 15.0 | 8.9 | <0.0001 |

**Table 2. Weighted participant characteristics for LDL cholesterol in Sample 3**

| Characteristic | Depression | Non-depression | *P* |
| --- | --- | --- | --- |
| Gender (%) |  |  | <0.0001 |
| Male | 37.0 | 49.7 |  |
| Female | 63.0 | 50.3 |  |
| Age (%) |  |  | <0.0001 |
| 13-39 | 36.8 | 43.2 |  |
| 40-59 | 38.5 | 30.9 |  |
| ≥60 | 24.7 | 25.9 |  |
| Race (%) |  |  | 0.0002 |
| Mexican American | 7.8 | 8.75 |  |
| Non-Hispanic White | 63.0 | 67.6 |  |
| Non-Hispanic Black | 7.2 | 10.9 |  |
| Other Hispanic | 15.8 | 5.2 |  |
| Other Race | 6.2 | 7.5 |  |
| Educational level (%) |  |  | <0.0001 |
| Less than 9th grade | 24.6 | 14.0 |  |
| 9-11th grade | 1.4 | 1.4 |  |
| High school graduate | 26.3 | 23.4 |  |
| Some college or AA degree | 35.1 | 30.6 |  |
| College graduate or above | 12.6 | 30.6 |  |
| Poverty income ratio (%) |  |  | <0.0001 |
| Poor | 30.6 | 13.6 |  |
| Near-poor | 27.6 | 19.9 |  |
| Middle-income | 22.0 | 30.0 |  |
| High-income | 19.8 | 36.4 |  |
| Marital status (%) |  |  | <0.0001 |
| Married/Living with partner | 47.5 | 63.7 |  |
| Divorced/Widowed | 23.7 | 14.0 |  |
| Never married | 22.4 | 20.3 |  |
| Separated | 6.5 | 2.0 |  |
| Alcohol status (%) |  |  | <0.0001 |
| Never | 11.1 | 11.3 |  |
| Former | 21.5 | 14.0 |  |
| Current | 67.5 | 74.7 |  |
| Smoking status (%) |  |  | <0.0001 |
| Never | 37.7 | 56.5 |  |
| Former | 24.2 | 25.8 |  |
| Current | 38.1 | 17.7 |  |
| Health status (%) |  |  | <0.0001 |
| Excellent | 1.9 | 11.1 |  |
| Very good | 12.2 | 34.2 |  |
| Good | 34.5 | 40.6 |  |
| Fair | 38.2 | 12.7 |  |
| Poor | 13.3 | 1.5 |  |
| BMI (%) |  |  | 0.0916 |
| Low | 2.8 | 2.0 |  |
| Normal | 28.4 | 32.4 |  |
| Overweight | 68.8 | 65.6 |  |
| Physical function score (%) |  |  | <0.0001 |
| 0 | 29.4 | 44.7 |  |
| 1 | 16.0 | 20.2 |  |
| 2 | 12.1 | 13.0 |  |
| 3 | 10.9 | 9.6 |  |
| 4 | 13.4 | 6.2 |  |
| 5 | 11.6 | 4.1 |  |
| 6 | 6.6 | 2.2 |  |
| Comorbidity Stroke (%) | 6.1 | 2.8 | 0.0002 |
| Comorbidity coronary heart disease (%) | 6.3 | 4.1 | 0.0240 |
| Comorbidity cancer (%) | 9.3 | 9.4 | 0.9662 |
| Comorbidity high blood pressure (%) | 44.4 | 30.6 | <0.0001 |
| Comorbidity diabetes (%) | 15.0 | 9.8 | 0.0039 |
| Take prescription for high cholesterol (%) | 26.9 | 25.4 | 0.4927 |
